# *“That Pregnancy Can Bring Noise into the Family”:* Exploring Intimate Partner Sexual Violence during Pregnancy in the Context of HIV in Zimbabwe

**DOI:** 10.1371/journal.pone.0043148

**Published:** 2012-08-24

**Authors:** Simukai Shamu, Naeemah Abrahams, Marleen Temmerman, Tamara Shefer, Christina Zarowsky

**Affiliations:** 1 School of Public Health, University of the Western Cape, Cape Town, South Africa; 2 Gender and Health Research Unit, Medical Research Council, Cape Town, South Africa; 3 International Centre for Reproductive Health, Ghent University, Ghent, Belgium; 4 Women's and Gender Studies, University of the Western Cape, Cape Town, South Africa; Vanderbilt University, United States of America

## Abstract

**Background:**

Globally, studies report a high prevalence of intimate partner sexual violence (IPSV) and an association with HIV infection. Despite the criminalisation of IPSV and deliberate sexual HIV infection in Zimbabwe, IPSV remains common. This study explored women's and health workers' perspectives and experiences of sexuality and sexual violence in pregnancy, including in relation to HIV testing.

**Methods:**

This qualitative study was part of a larger study of the dynamics of intimate partner violence and HIV in pregnancy in Zimbabwe. Key informant interviews were conducted with health workers and focus group discussions were held with 64 pregnant or nursing mothers attending antenatal and postnatal care clinics in low-income neighbourhoods of Harare, covering the major thematic areas of validated sexual violence research instruments. Thematic content analysis of audio-recorded and transcribed data was conducted.

**Results:**

While women reported some positive experiences of sex in pregnancy, most participants commonly experienced coercive sexual practices. They reported that men failed to understand, or refused to accept, pregnancy and its associated emotional changes, and often forced painful and degrading sexual acts on them, usually while the men were under the influence of alcohol or illicit drugs. Men often refused or delayed HIV testing, and participants reported accounts of HIV-positive men not disclosing their status to their partners and deliberately infecting or attempting to infect them. Women's passive acceptance of sexual violence was influenced by advice they received from other females to subordinate to their partners and to not deprive men of their conjugal sexual rights.

**Conclusions:**

Cultural and societal factors, unequal gender norms and practices, women's economic vulnerability, and men's failure to understand pregnancy and emotional changes, influence men to perpetrate IPSV, leading to high risk of HIV infection.

## Introduction

Gender-based violence in general, including coercive sexual practices, is widely understood as an expression of male control and domination over women [1], [2], [3]. Although non-consensual sex both in marriage and dating relationships is common, it is believed to be under-reported [4]. Nevertheless, research conducted to understand unsafe and inequitable sexual relationships in the light of the high rate of HIV shows that coercive sexual practices, from more subtle forms to physical rape, are endemic in heterosexual intimate relationships in southern Africa [5], [6], [7]. In South Africa, for example, in a 2008 study conducted in the Eastern Cape and KwaZulu-Natal, nearly one third of men (28%) admitted to having raped a woman [8].

One of the challenges in studying intimate partner violence is the definition of sexual violence, which includes a spectrum of actions that vary from non-physical persuasive language to the use of physical force [9]. Definitions of sexual violence in southern Africa include: penetrative sex without the partner's agreement, enacted by means of verbal pressure or physical force which may include emotional manipulation, threat, trickery, verbal persistence or not taking ‘no’ for an answer; being locked in a room; and being physically assaulted [9], [10], [11], [12]. Njovana and Watts [13] include forced pregnancy as another dimension of sexual abuse in their study in Zimbabwe. In South Africa, young women report ‘giving in’ to male pressure for sex because of ‘love’, commitment and fear of losing the relationship [14], [15], [16].

In most southern African countries, rape has been redefined from the limited common law definition whereby it consists of only penal-vaginal sexual intercourse without a woman's consent [17] to also include acts of non-consensual sexual penetration with the penis, finger or object into the vagina, anus, or mouth of another person; and other non-penetrative sexual acts against one's will. This study acknowledges the continuum of violence and the importance of humiliation, degradation and violation of a woman's sexual integrity. More subtle, non-physical forms of coercion in which a partner consents to sex when they do not want it, due to power inequalities and normative roles and practices, are also viewed as sexual violence.

Njovana and Watts [13] and Osirim [18] argue that in Zimbabwe, the Shona culture and the economic downturn respectively, have perpetuated unequal gender relations that increase the risk of intimate partner violence. Regarding gender socialisation and Shona culture, Kambarami [19] argues that at puberty, girls are taught how to please their future husbands as well as to be gentle, submissive and obedient wives. This furthers gender inequality that arguably perpetuates intimate partner sexual violence [20].

Gender discrimination and female subordination in Zimbabwe is historical and was strengthened by colonial administrative policies which subordinated women. The colonial period saw the codification of customary practices into a rigid draconian law that discriminated against women, including officialising male control over women's sexuality. For example, the authority over a single woman's sexuality, sexual and fertility rights was transferred from fathers and brothers to husbands upon marriage, hence putting a woman's lifetime under the control and subordination of men [18], [21]. In addition, the influence of the marriage institution and the church is also viewed as encouraging male domination of female sexuality. It is in this broader environment of male domination of female sexuality, sexual and fertility rights that women continue to experience sexual violence in partnerships. Although some policies against gender inequality and violence such as the Domestic Violence Act and Sexual Offences Act have been instituted in the post-independence period, implementation of these policies has not been adequate.

The last decade has been marked by an increasing acknowledgement of the role of normative gender roles and power inequalities in HIV/AIDS [20] and exploring the interconnections between gender-based violence (particularly inequitable and coercive sexual practices) and HIV infection [1], [22], [23]. Zimbabwe is one of the few countries in the world that has criminalised marital rape in the context of HIV infection [17], [24]. The law also gives women power to seek protection orders against their violent partners. Despite this, up to a quarter of Zimbabwean women of child-bearing age report sexual abuse, with most (65%) of the abuse taking place in intimate relationships, and at least 8% occurring during pregnancy [25], [26], [27]. Although the criminalisation of marital rape has been operational since 2001, according to Chirawu [21], up until 2006, no perpetrator had ever been prosecuted for marital rape. Underreporting of domestic violence has been widely noted in South Africa with respect to both formal police record-keeping and in epidemiological studies [4]. Research with the Musasa, a non-governmental organisation working against gender-based violence in Zimbabwe, showed that it took some women up to 10 years to seek help after the abuse [13].

Sexual violence or rape during pregnancy has not attracted as much research in Africa as elsewhere in the world. Many studies subsume reports of its prevalence and effects under the broad term of ‘physical violence’. The prevalence of sexual violence during pregnancy in Africa was reported in a systematic review, to range from 2,7% to 26,5% [28]. The protective effect of pregnancy on coercive sexual practices has been recorded in both western and non-western societies, although at different reporting periods. In China for instance, the prevalence of coercive sexual practices declined from 5,8% in the 12 months before pregnancy to 2,8% during pregnancy, before it rose again to 4,9% after pregnancy [29]. In Belgium, coercive sex was higher (0,9%) in the 12 months before pregnancy and lowered significantly (0,2%) during pregnancy [30]. Most of these studies were conducted in low HIV prevalence communities; the situation could be different in Zimbabwe where HIV prevalence is one of the highest in the world. Women who test for HIV and seek HIV preventive mechanisms such as condom use may experience coercive and unprotected sex from their partners who question the idea of using condoms in marriages.

Since the enactment of laws in Zimbabwe against partner rape and deliberate STD/HIV infection in intimate relationships there has not been a dedicated study to explore current experiences of sexual violence. HIV testing among antenatal care attendees in Zimbabwe shifted from a patient-initiated model based on the “opt-in” approach to a provider-initiated testing and counseling model in which all antenatal care attendees are invited to test and only those who “opt out” will not be tested [27]. This model, sometimes known as the “opt-out” approach, has significantly increased HIV testing coverage from 65% to 99.9% [27]. These changes in policy related to partner rape and HIV testing in public health facilities are both important to assess in their own right, and provide a framework within which to assess and interpret intimate partner sexual violence (IPSV) and how it intersects with HIV. There is a need to provide an in-depth understanding of the HIV risk factors that are increasingly documented – but with little contextualization - in quantitative research. The aim of this study was to explore participants' experiences of IPSV during pregnancy, including after HIV testing, with a particular focus on how such violence may interlink with HIV infection.

## Methods

A qualitative study on IPSV during pregnancy and HIV testing was conducted using focus group discussions (FGDs) involving pregnant and nursing mothers and in-depth interviews with health workers. Qualitative thematic analysis was used to develop a theoretical understanding of sexual violence experiences during pregnancy and their coexistence with HIV infection, testing and disclosure. Data were collected at six public primary health care facilities (pre and post-natal) in low-income high density residential suburbs in Harare in April and May 2010. This exploratory study informs a larger mixed method study of the prevalence and dynamics of IPSV and HIV in pregnancy, aimed at developing interventions with health workers, women and men against IPSV.

### Ethics

The study was conducted following the WHO Guidelines on researching violence against women [31]. The Medical Research Council of Zimbabwe and the University of the Western Cape ethics committees approved the study. Permission to conduct the study was granted by the Harare City Health Directorate. All study participants were given full information about the research and its aims. All participants voluntarily gave written consent to participate in the study. Participants were assured that they could leave the research at any time. Confidentiality and anonymity were maintained by asking participants not to mention their names or disclose their HIV status during focus group discussions.

### Focus group discussions (FGDs)

Seven FGDs were held in Shona with 64 women at six public health facilities. Four FGDs involved pregnant women attending second or third trimester antenatal care (ANC) clinics and three were held with nursing mothers attending postnatal care (PNC). All women were reported to have tested for HIV during their initial ANC visits through the provider initiated HIV testing and counseling (PITC), although we did not independently verify their individual test results.

Pregnant and nursing mothers were aged between 18 and 38 years old. Most were not formally employed. Almost all reached or had completed 11 years of formal education. Half were either carrying first pregnancies or had one child. The highest number of pregnancies a participant had ever had was four. Almost all reported that they were currently married or cohabiting.

FGDs, led by a researcher and a trained research assistant, lasted 1 to 1½ hours. All were audio recorded and field notes were also taken. The FGD Guide covered the major thematic areas contained in the validated sexual violence research instruments designed by the WHO [23] which are being used in the broader study.

The initial questions focused on women's household chores, planning in the household, and likes and dislikes during pregnancy. The discussions eventually progressed into the more sensitive issues of sexual violence and HIV. To enable open discussion, participants were invited to narrate their experiences as stories about other people or “someone I know” and not to feel obliged to disclose their own personal experiences. This technique of disclosing sensitive personal information in third person was used in a sensitive study of HIV and risky sexual practices among young female university students in South Africa [32]. Our use of this technique helped to increase rapport and disclosure of sensitive sexual violence experiences which would not have been shared as personal experiences.

### Interviews with health workers

Seven key informant interviews were conducted with health workers (six nurse midwives and one HIV testing nurse) at six facilities. These face-to-face open-ended interviews were held privately with participants in English by a fluently bilingual (English-Shona) researcher, but participants often switched to Shona (the local language) when quoting verbatim from their recollections of encounters with abused women or around disclosure issues since nurses communicate with clients in Shona and not in English. Data were captured by audio recording and written notes. The information collected from health workers was then cross-checked with that collected from FGDs, to understand the organisation and impact of antenatal and postnatal care, provider-initiated HIV counseling and testing, and disclosure of HIV results.

### Data analysis

Audio recorded data was transcribed verbatim and vernacular text was translated to English – translations were checked for accuracy, consistency and validity. Information from field notes was also cross checked with information from the tapes before coding. All scripts were loaded into OpenCode qualitative software to organise the data into codes and categories. During this process, transcripts were repeatedly read and codes constructed based on the research objectives. Common themes were formed and new codes formulated as themes emerged. We systematically followed this process of coding and categorizing the data under themes presented in the findings section until we were satisfied that all data that fit the themes were relevantly coded. Thematic content analysis was used to analyse the data in each theme. We assessed the content and meaning of the information in each theme in line with the study objectives as well as findings from other studies. Whilst the concept of analytic induction was used to examine similarities between information from FGD participants and that from health workers, differences were also noted. The findings section below presents each theme.

## Results

The first significant finding was that all participants discussed the issues openly and both health workers and participants reported coercive or violent sex during pregnancy as commonplace yet complex. Results are presented by first describing social norms around pregnancy. Next norms relating to sexual relations in marriage are outlined. Coercive sexual practices that participants reported are then described, as well as reports of positive and pleasurable sexual experiences. Finally, we report how respondents discussed the issue of sexual relations around the time of HIV testing and disclosure.

### A. Social norms around pregnancy and expectations of child-bearing

Participants reported that women have less decision making power than their partners concerning their reproductive health and when to fall pregnant. Many first pregnancies were not planned and this was viewed as a facilitative factor in partner conflict and violence during pregnancy. For example, some young men raped their partners during dating, and these women later became their wives. Participants reported how date rape with the intention of impregnating a girl was carried out if the girl refused a marriage offer, and was generally perpetrated by poorer men who lacked the money for bride wealth. According to both FGD participants and key informants, these unintended pregnancies usually led to violence later in the relationship, with some relationships ending during pregnancy. One pregnant woman remarked, *“That pregnancy can bring noise into the family…l think it all depends on how the pregnancy came about.”* (FGD Pregnant Women, Facility D).

Participants also reported a widespread practice of family control over their reproduction, with relatives, especially from the man's side, compelling the woman to have a baby:

*“Some mothers are pressured by their parents but most of the time the pressure comes from the men's side. They will start saying that you did not come here [to our family] to eat [but rather you have a duty to bear children for the family]. You notice how they [in-laws] speak, that they now want you to have another child.”* (FGD Pregnant Women, Facility F).

At times, for fear of being accused of intervening in a couple's private life, in-laws and aunts *“speak in riddles and parables”* telling the daughter-in-law to become pregnant. In the case of those with children already, in-laws *“may take their [the couple's] last baby to their rural area which traditionally means the child is disturbing them from having another baby”* or may come to town saying, *“they wanted to be spoiled”* by their son who does not have a child. A few participants reported how in-laws and aunts directly advised a newly-married wife to become pregnant lest she be accused of barrenness.

### B. Norms relating to marital sexual relations

Another key finding was the importance of norms relating to marital sexual relations. Women got advice about sexual intercourse during pregnancy from many sources. Tradition and the institution of marriage emerged as major factors impacting on a pregnant woman's capacity to resist sexual violence and for men to justify their perpetration thereof. Despite not agreeing to have sex, women reported that they had sex to please their partners according to tradition. One woman in a FGD at Facility B mentioned that, *“When you are married you shouldn't refuse [sex]”*. Another said, “… *in our tradition it's not possible [to deny him sex]*. *You must just pretend you are enjoying it by making the necessary noise in bed”.* To these women, sex was a matter of fulfilling the traditional role of being wife, which dictates that a man has rights over the sexuality of his wife. If she refuses sex she could be punished bitterly, for example, by being chased away from home. As one woman narrated, *“Yes he will tell you that it's his right and it's his house. He will tell you to get down from the bed. If you get down he will tell you to leave his house. So you will see that if you go out it will be difficult to come back. Therefore, you end up doing it because you would have been forced.”* (FGD Pregnant Women, Facility E).

For many women, saving a marriage by observing a husband/partner's demands was an important aspect of womanhood. At Facility D, one older woman reprimanded a young expecting mother, saying, *“Sometimes we just have to understand and try and save our marriages. You can have sex once or twice per week to protect your marriage. We cannot encourage each other such bad habits of refusing sex. I do not think it is a good idea.”*(FGD Pregnant Women, Facility D). The younger women disagreed, while the older women supported this view.

The practice of paying bridewealth in Zimbabwe also facilitates the domination of female sexuality by husbands as anthropologists argue that bridewealth transfers control of female sexuality from a woman's family of origin to her husband during marriage [19], [21], [25]. In a FGD at Facility E, women overwhelmingly highlighted bridewealth as a major contributor to forced sex. They interpreted bridewealth as giving a married man unlimited access to sexual intercourse with his wife, making it difficult for women to refuse sex. The following extract shows how men also reportedly take advantage of bridewealth to demand sex:

Respondent 1: Some men will just hide behind the fact that “I married you” and when I am pregnant (interruption: Yes! All agreeing) so I end up doing it. He shouldn't be denied.
Respondent 2: This issue of lobola (bride wealth)! (interjected by laughter)
Respondent 3: This issue of lobola saying “I paid for you!” (FGD Pregnant Women, Facility E).

Participants reported that aunties advised women not to refuse their partners' sex, and thus played a major role in reinforcing women's inferior position:

*“They tell you not to deny him. If he becomes promiscuous [because you denied him sex] it will stress you more. So you end up forcing yourself to do it. You will pretend as if you like it.”*(FGD PNC Women, Facility A).

The reinforcement of women's submission to men's sexual demands extends well beyond contexts obviously and directly related to marital sexuality. Influence from broad-based social institutions, such as the church and the clinic, was also cited as manipulating women to tolerate forced sex. The role of health workers should not be underestimated. Pregnant women were counseled that they should not refuse or resist sex until they delivered, and they appeared to take this advice quite seriously. One woman explained how, during the health education talks at the antenatal clinic, the tradition of not refusing your husband sex under any circumstances was reinforced:

*“We came yesterday and the nurses taught us not to refuse our husbands sex because they will go out to small houses. Even when you feel you don't like it just do what you can so that you keep him satisfied. Try to push until labour. These are some of the teachings that you will not be aware of. They said breathe with two entrances [orifices] (Laughter). Some say at six months l will no longer have sex. Do not be fooled just try and give him sex so that he will be satisfied. As for me when l came from the clinic I changed at once. I am now doing what l can and not to deny him totally.”* (FGD Pregnant Women, Facility D).

Regarding the church, participants stated:

*“At church we were taught that you should not sleep facing opposite directions”* and that if one is in great pain they insisted that they were taught to *“just romance or do something different and not to deny him totally.”* (FGD Pregnant Women, Facility D).

However, some women reported that allowing men to do non-penetrative sex acts would eventually led these men to ask for or actually forcing sex.

### C. Coercive sexual practices

The majority of participants reported enduring coercive sexual practices during pregnancy, as they felt powerless to resist. Most expressed pain, displeasure and dislike for sex during the third trimester, and referred to uncomfortable sexual practices, having sex to keep the husband in the house, and social norms pressuring them to tolerate forced sex.

#### *i.* Uncomfortable and painful sexual styles and positions

Most participants reported that in their last trimester their husbands insisted on uncomfortable sexual acts against their will. These were commonly reported as, painful sexual positions, vigorous and energetic movements during sex, and sexual styles dictated by men for their personal satisfaction. These acts became even more painful closer to the delivery date as, “h*e would do it the way he likes not what I suggest…”* (FGD PNC Women, Facility F). Many participants reported being forced to perform styles they thought were degrading:

*“These men are very promiscuous and they want the styles that they get out there. Sometimes you will not be able or you won't know it (Interjection: he will be knowing plenty of styles!)* (They all burst into laughter). *Yes, they will be knowing plenty of them.”* (FGD Pregnant Women, Facility E).

Many participants admitted that they would rather have painful and unsatisfying sex to make their partners happy, rather than risk their partners having sex with other women if they refused them sex (which is often threatened if the wife refuses sex). Some pregnant women have learnt to tolerate painful sex, whilst others have used the physical exercises offered at the clinic to enable them to perform sex with limited pain. Women described both the painful positions and the exercises matter-of-factly:

*“Which is better getting the pain and boredom for thirty minutes whilst doing it than for him to go look for someone else? So you just do the position that he wants…And it will be over. Maybe he wouldn't want it every day. He will be happy saying that my wife is compromising. So you as a woman you just have to be strong. Isn't it that we have to be strong?”* (FGD Pregnant Women, Facility F).

Participants reported that men believed that having sex helps to clear conflict or anger. For example, they reported being forced to have sex after an argument in the hope that the wife will forget the misunderstanding. A man who beat his partner and then demanded sex from her reportedly argued, *“it is us, not our sexual organs, who have misunderstood each other.”* (FGD PNC women, Facility F). In other FGDs, participants reported similar coercive sex, *“Even when l am angry and l have crossed my legs the husband will try to open my legs.”* (FGD Pregnant Women, Facility D).

#### *ii.* Having sex to lure him away from multiple sexual partnerships

Participants across FGDs reported that the desire to keep their partner ‘in the house’ – or to return to the house – led them to accept coerced sex, no matter how painful or unwilling they were. They reported that their partners had ‘small houses’ (a term used in Zimbabwe to refer to girlfriends/partners other than the main partner) and that they would endure sex in order to ensure that their partner did not take on other sexual partners:

*“…sometimes you won't be interested but you just force yourself to do it…You would have been told [by nurses and aunties] not to deny him. Otherwise he will be promiscuous.*

*You will just do it to satisfy him. As for me even if I don't feel like it I just force myself to do it just to make him happy. If I don't do that he will leave me and go find someone else to sleep with. So if he does that and I hear about it or see it, it will be very painful for me.”* (FGD PNC Women, Facility A).

Nurses confirmed that they heard stories of women reporting their partners as being philanderers during their pregnancy. An HIV counsellor in the maternity clinic reported: *“we hear women saying that, “our husbands prostitute”, “he is not sleeping at home”, “he is again in love with his ex-lover…”* (Interview with Counsellor, Facility E).

Some participants reported that if they go for a long time without sex, the husband will become suspicious and accuse her of promiscuity; and it was therefore better to accept sex despite feeling unwell.

#### *iii.* The effect of pregnancy and emotional changes on sexual violence

The pre-pregnancy period was reportedly characterised by positive sexual experiences since women could perform painless sex and were generally in good health. Participants reported that the emotional changes that took place during pregnancy, which often resulted in women wanting sex less frequently, were not well understood by men. This sharp contrast in sexual relations between the pre-pregnancy and pregnancy period sparked conflict between partners but inevitably ended in men forcing their partners to have sex even though the woman was in pain or felt ill.

*“What I have noticed is that men always want sex more when you are pregnant than when you are not.”* (FGD Pregnant Women, Facility F).

*“Even if you say you are not feeling like doing it he will be saying his temperature and your temperature those days will be suiting each other. So he will force you.”* (FGD Pregnant Women, Facility E).

*“Sometimes you will be feeling that you can no longer do it. Sometimes wanting it once or after two days. But he will be expecting it every day. That's being cruel because your body can no longer sustain that. It will be affecting you and it's painful.”* (FGD Pregnant Women, Facility E).

Other participants reported not wanting sex at all during pregnancy even though they used to like it in the pre-pregnancy period. Some women commented that their partners would be more vigorous, leaving them in great pain after sex:

*“When a woman is pregnant you will not even want to have sex but it causes problems. Husbands do not understand because they do not consider that I am now in a different situation, even if I used to like sex all the time if they will remember that when you were not pregnant that's not what you used to do. As for me l only think about my husband when he is not around.”* (FGD Pregnant Women, Facility D).

Many participants reported feeling too ill and weak to have sex in late pregnancy, yet men interpreted this as dislike for sex and ‘uncultural’ and so forced their partners to have sex.

Participants believed that pregnancy and emotional changes varied with the sex of the baby and whether the pregnancy was the first or a subsequent one. They associated the boy child with more problems from the partner, their dislike of sexual intercourse, and painful sex; whilst the girl child was associated with women ‘nagging’ the partner for more sex. The same information was reported by participants who had given birth before:

*“It varies across pregnancies. My current pregnancy is different from all the previous. When I was pregnant with my daughter, it was me who was nagging him; asking for sex even during the day. He would say, ‘Can't you see your tummy is now too big’. I would tell him that was not his concern. With my son's pregnancy we never agreed (to have sex) till I delivered. It depends with the pregnancy. Like now I feel it's painful. I feel like I am cracking between my legs but I will just say what else can I do.”* (Laughing). (FGD Pregnant Women, Facility F).

*“It happened when I was pregnant with this child. The pregnancy carrying this boy was usually troublesome. I ended up leaving him (going) to my family…”* [The Shona custom called kusungira requires that a pregnant woman spends the third trimester with her mother, learning to become a mother but this woman left the husband much earlier because of violence] (FGD PNC Women, Facility F).

Participants reported that the influence of drugs also led their partners to engage in vigorous, painful and uncaring sexual acts:

*“It's like when a man forces you to sleep with him or you have agreed to sleep with him on that occasion. He might come drunk or after having smoked dagga [marijuana]. (Laughing). He will be planning to fix you (Laughing). It will be very clear that even if he says let's sleep together you will refuse. Maybe because the last time you slept with him (in that state) you really felt your back aching. So this issue of being forced to sleep with him is very bad”* (FGD Pregnant Women, Facility C).

### D. Positive, equitable and pleasurable experiences of sexuality in pregnancy

A number of participants reported positive and consensual sexual experiences during pregnancy. Some participants spoke about how their husbands understood their loss of sexual desire during pregnancy, however in some cases it was a challenge to ensure such understanding. As one pregnant woman remarked, *“I think someone explained to him because he now understands”*. The following statement illustrates this further:

*“As for me l can give him when l feel like giving him when l see that many days have passed without sex. We even joke about it with my husband. He sometimes phones me before he comes home and asks me if we were going to have sex. I will then tell him if the baby desires to have his daddy or not. Then he will come home fully aware that we will sleep facing opposite sides. To me that is respect.”* (FGD Pregnant Women, Facility D).

In some FGDs, women reported that they participated in ‘kitchen parties’ where they shared experiences and information about sexual matters in their relationships, including during pregnancy with the aim of making sex pleasurable even in difficult circumstances. [A kitchen party in urban Zimbabwe is a social gathering organised and attended by women only, originally to present kitchenware gifts to a newly married woman but has now extended to advising a newly married woman about sex and sexuality in a marriage]. These educational forums reportedly included information about women's rights to decide, initiate, lead in sex, and experience sexual pleasure.

It emerged that some women desired more sexual intimacy during pregnancy and in some cases their desire outstripped that of their partners. They would regularly initiate and even demand sex. This led to instances where they felt they were ‘forcing’ men to engage in sex more frequently than they otherwise might have wanted. The two statements below illustrate how women exercise power and agency over sexual matters in their relationships.

*“My husband… said his friend was coming to work very tired every day…he said that the wife to his friend demanded sex every day saying that the nurses have said so until delivery (Laughter). The pregnancy wants the father but the father does not want the pregnancy. Thus the husband is now doing it for duty so some husbands are being forced to have sex too.”* (FGD Pregnant Women, Facility D).

*“It all depends on one's feelings. Some might want it so many times. In most cases, it is the woman. Sometimes we discuss this as women. Some women can really stand their ground. They can go on for longer hours. Much more than the husband! Some husbands are in a tight situation such that they don't even sleep at night. She will be constantly waking him up wanting more rounds of sex…”* (FGD Pregnant Women, Facility A).

Again, the gender of the baby was believed to impact on sexual desire. In a group of post-partum mothers, participants who were pregnant with girls reportedly desired more frequent sex and found sex more pleasurable during pregnancy, than those who had been pregnant with boys. The same was also reported by some participants who compared their previous pregnancy with a boy child to their pregnancy with a girl child.

### E. The effect of HIV testing on sexual experiences and relationships

Participants mentioned a number of control issues, abuse and sexual relationship issues related to HIV testing and prevention of mother-to-child transmission of HIV. These included needing a partner's permission to seek reproductive health care, men's refusal to test for HIV, refusing condom use to prevent HIV, refusing to disclose HIV results to female partners, and intentionally trying to infect a wife with HIV.

The study found unequal gender relations regarding HIV testing as men refused to test but expected to infer their HIV status from their partners' results. Participants and health workers reported that men saw having a baby as a way of knowing their own HIV status through their partners' HIV tests at the ANC.

*“When suspicious of HIV, men do not usually test through the needle but through having a baby, knowing that the wife will be tested at the clinic…* (HIV Counsellor, Facility E).

Many participants reported that after they had tested for HIV and received HIV education, they requested that their partners also test. Many refused, claiming that if the woman tested negative, then he was also negative as they were having unprotected sex. Other men lied that they had tested at work. Many refused to disclose their results to their partners and subsequently perpetrated sexual violence. Stories of intentionally infecting a female partner after the man tested positive were common in FGDs:

*“The husband might know that he is sick so he will come and force his wife to sleep with him and he infects her. There are men like that who do not tell their wives the truth about their status. You will only find out when you come this side [PITC clinic] that you have HIV.”* (FGD PNC Women, Facility A).

*“The husband was sick (TB) and the wife looked after him and he recovered. Now after some years the wife got pregnant and the husband was the one who forced the wife to have a child knowing very well his experiments he was doing. The wife tested HIV positive…She persuaded him [to test] … and they were both positive… and the husband took that to say the wife was the one who had brought the disease into the house.”* (FGD Pregnant Women, Facility D).

*“What we have discovered is that some of these mothers when they test positive…later on you discover that this man had earlier on tested and he knew his status but did not disclose it to the partner… She will come and tell me, ‘Sister I don't understand this. When I told my husband about my status, he was not surprised at all…I think he tested before but he did not tell me about it’”* (Interview with Sister in Charge, Facility F).

In the case of a woman testing positive, the male would often accuse her of prostitution and of *“bringing the disease”* into the house. If the man tested positive he would still blame the woman for infecting him. Some participants also reported that they risked being chased away from home if they tested positive.

Some women who tested negative tried to refuse their partners sex until they were tested. However, they eventually succumbed to his pressure:

“*I asked my partner to go for a test but he refused. He said he does not have the disease saying that since I didn't have it meant that he didn't have it as well. I told him to go for tests. I even refused to sleep with him… if he was not tested. In the end I gave in because he was not going for the tests anyway”* (FGD PNC Women, Facility A).

Other women who tested negative requested their partners to use condoms until they were tested. They would often face resistance, as described below:

*“There is another problem when the wife is negative the husband will do tricks so as to make the wife positive too. There is a couple l know whereby the husband will take off the condom during sex in an attempt to infect the wife. The wife tested negative three times when these incidents occurred and she decided to leave the husband… Yet she was the one who was looking after him and going to collect his medication.”* (FGD Pregnant Women, Facility D).

*“You would like him to use a condom because you will be aware of how things stand. The man won't accept that.”* (FGD Pregnant Women, Facility E).

*“Another form of abuse that we see is that men refuse to use condoms when they actually know that they are positive.”* (Interview with Counsellor, Facility E).

## Discussion

The major theme of the narratives recounted by the participants was men's use of overt or threatened violence or abandonment to control female sexuality, and norms that undermine women's control over their sexuality. A related theme was that HIV testing and disclosure increases the risk of sexual coercion and violence, while sexual coercion and violence limit women's – and men's – capacity to protect themselves from or appropriately manage HIV infection. The paper also highlighted that women's diminished sexual interest during pregnancy, especially in the third trimester, was not understood or respected by men. However, the women do not recount a simple and uniform passivity or “victimhood”: we also presented evidence of how women demonstrated agency in negotiating and demanding pleasurable and equitable sexual relations during pregnancy.

Underlying the theme of physical and emotional changes during pregnancy and how this affects sexuality is the importance of social norms, unequal power relations, and the social and economic vulnerability of women as reported elsewhere [33]. Familial and cultural norms around the role of married women, reproductive health and pregnancy, and when and how sex is to be performed, impact on women's agency and facilitate a situation which is conducive to sexual violence.

Women's economic vulnerability further facilitates partner abuse, in line with other studies [3], [28], [34]. Almost all participants in the study were unemployed and financially dependent on their partners for their (and their child's) survival and support during and after the pregnancy. Given the harsh economic environment in Zimbabwe at the time of this research, where unemployment was around 80%, risking the loss of a marriage or partnership with an employed man during pregnancy was detrimental to economic survival and meeting health needs. The burden of sexual violence was lighter for many women than the perceived economic burden of being divorced. Respondents expressed an inability to refuse coercive and unsafe sex for fear of being divorced in a community in which single women have less moral worth and are exposed to economic vagaries, compared to married women. This relationship can be compared to the patron-client relationships which adolescents in East Africa entered with ‘sugar daddies’ (older sexual partners) in return for economic gifts from these abusive partners [35].

‘Culture’, ‘tradition’ and social institutions play a major role in initiating, strengthening and reproducing women's subordinate position and the potential to be abused by their partners. The widely taught traditional norm of sexual submission of wives was reinforced by the family, church and health institutions. Von Sydow's [36] review of 59 studies of sexuality during pregnancy notes that most participants reported that health workers' advice about sexual intercourse was restrictive. Our findings suggest that health workers, who share the same culture as their clients, subscribe to the same doctrine of male control over women's sexuality. This calls for widespread community campaigns and education of health workers for gender equity in sexuality.

Traditional feminine and masculine roles regarding sex and sexuality apply to pregnant women as much as they do to women in general. In other anthropological studies in Zimbabwe, women were reportedly not expected to initiate sex or show sexual pleasure as this suggests sexual experience, whereas they are expected to be less sexually experienced than their partners [18], [19]. However, some participants in our study openly discussed their sexuality and heightened sexual desires during pregnancy, foregrounding their sense of agency in their relationships. It is especially interesting to compare this with the majority of studies in southern Africa that highlight women's vulnerability and unequal power relationships with men, as well as the absence of a positive discourse about women's sexuality (see for example [37], [38], [39]).

Negative forms of masculinity were also demonstrated in the study with men perpetrating risky sexual behaviours such as having multiple sexual partnerships; forcing sex; denying that they could be HIV infected; refusing HIV testing or safe sex; not disclosing their HIV status; blaming partners for positive test results; and perpetrating sexual violence under the influence of alcohol. Similar heterosexual masculinity amongst South African men have been reported elsewhere [20], [40].

Although there are laws in Zimbabwe that prohibit marital rape and intentional HIV infection in partnerships, the practice remains common, and is characterised more by non-physical coercion than by physical force. Unlike violence perpetrated by strangers which involves physical force [41], rape in this study is mainly marital and stems from women's cultural submission to men. Women learned and increasingly felt that they had to perform ‘wifely duties’ by being obedient to their partners' sexual demands. In most cases women reported feeling obligated and ‘forced themselves’ to have sex to please their partners, not themselves.

We reported on women's agency as demonstrated by some women who were empowered and could negotiate or dictate more equitable and satisfying sexual relationships. This agency shows that women were not just passive sexual partners; they also demonstrated some form of sexual power over their partners during pregnancy. This finding resonates with discussions of changing notions of empowerment that Silberschmidt [42] postulated among women in East Africa in which women increasingly gained sexual power over their economically disempowered men.

The study had some limitations. The sample was small and each participant had only one opportunity to reflect on and discuss the issues. Most of the data is based on women's reported experiences which may not necessarily reflect their partner's views and behaviours.

More research involving men is needed to understand their views on the perpetration of sexual violence. However, studies on violence against women show that it is very unlikely for women to over-report their experiences, and that in fact they tend to minimise the violence [4].

## Conclusions

This paper has shown how complex sexual violence during pregnancy is in Zimbabwe and the many ways in which it is shaped by traditional norms and reinforced by social institutions, kinship and professional relationships. Most of the reported sexual violence was in the form of coercive sexual practices influenced by dominant male masculinity in society. Whilst pregnancy is an opportunity to test and disclose HIV status [43], participants reported that an HIV positive result can lead to abandonment, divorce and sexual violence. Such experiences have been reported elsewhere [44], [45]. Furthermore, women's economic dependency is easily exploited by their partners, especially when women are at their most vulnerable – during pregnancy.

Educating communities about, and implementing multi-sectoral approaches towards, safe and equitable sexual relations are crucial steps to containing sexual violence during pregnancy. An important part of this is to financially empower women through educational workshops and credit schemes as effectively demonstrated by the Stepping Stones [34] and IMAGE [22] studies in South Africa. However, transforming unhealthy and coercive models of masculinity and femininity will require sustained efforts across all levels and institutions of society.
